# Bilharziasis and Its History

**Published:** 1919-07-12

**Authors:** 


### BILHARZIASIS AND ITS HISTORY.

### An Example of Recent Success.

Throughout the past decade so great have been the advances in our knowledge of many types of parasitic disease that one is often in danger of taking as complete and final the majority of accepted views on these conditions. Actually, if one goes deeply into the facts of each particular instance, it is found that many are lacking in exact detail of one or other etiological factor, or that if the life history of the parasite, the mode of human infection, or prophylaxis, be fully understood, then our known methods of treatment are found to fall far short of perfection. There is almost invariably some weak link in the chain, but, when research makes strides towards the completion of small but essential sections in the history of such disease and towards man's protection against its onslaughts, then, by tracing the whole story as it lies before us, we may enjoy one of the most fascinating chapters to be found in medical literature.

### Early Experiences.

A typical instance arises in connection with bilharziasis. For many years investigation of this disease, the major form of the parasite and a number of clinical facts have occupied the time of a whole series of workers. Even in 1870-1884 attempts were made by Cobbold, Sonsino and Lortet to trace the development of Egyptian bilharzia in intermediate fresh-water hosts, and to ascertain the exact mode of entry into the human system. Their efforts were not, however, immediately fruitful, and it was not until 1908-1913 that the earliest successful investigations into the morphology and pathology of the bilharzia worms were performed by a number of Japanese experts. They dealt with the species proper to their own country, *Bilharzia japonica*, and Fujinama and Nakamura were the first to prove definitely that normal animals could be infected from certain streams by mere immersion in the water. Then Matsuura found that a short time after he had simply waded up to the knee in these waters, he passed definite bilharzia ova in his stools. Finally, Miyairi and Ogata discovered and described both the fresh-water molluscs, which act as intermediate hosts, and the free swimming bilharzia larvæ, which are capable of infecting man. At this time Leiper and Atkinson were also carrying on investigations in Japan, and they, having definitely localised the host in a mollusc, *Katayama nosophora*, completed the history by infecting normal mice through the skin and demonstrating later both adult male and female bilharzia worms in the portal system of the same animals.

### The Egyptian Mission of 1915.

Thus at the outbreak of war we had a relatively complete knowledge of the Japanese species, and, realising the high endemic incidence of the corresponding disease in the Nile valley, and the grave risk incurred by the large bodies of troops stationed in this area, the War Office despatched a mission under Leiper to investigate Egyptian bilharziasis. Referring to the complete success of this mission, Dr. N. Hamilton Fairley, lecturer on parasitology to the Egyptian University, tells us that,

> Within six weeks of commencing work in February 1915, the non-eyed, bifid tailed cercariæ (the larvæ characteristic of the genus) were found in two different genera of snails. During the following year Leiper followed up his observations by completely vindicating the existence of two species of bilharzia worms in man. The one now known as *Bilharzia hæmatobia* presented distinct morphological features, laid a terminal-spined egg, and developed in a sinistral and spiralled fresh-water snail. The second is now known as *Bilharzia mansoni*. It gives rise to a *lateral-spined* egg, and develops in a flat fresh-water snail. In addition to this, he infected animals via the unbroken skin and via the alimentary tract.

But, apart from its being one of those diseases which the needs of war brought us so much nearer to understanding, there is great interest to be derived from the life-history of this parasite outside the human body, particularly as knowledge of this phase has given us a key to direct prophylaxis.

### The Extra-human Life Cycle.

The spined egg, whether passed with infected human urine or fæces, if it reaches water, hatches into a free-swimming larva or *miracidium*, which, left to itself, perishes in about twenty-four hours. It was with these *miracidia* that the earlier workers unsuccessfully attempted to produce re-infection in man, and the cause of their failure is now obvious. An intermediate host is necessary, and this, which, if found, the *miracidia* enter, is one of the species of fresh-water snail described above. In the body of the mollusc the larva becomes a sporocyst, which gives rise to a number of daughter sporocysts, and these in their turn produce enormous numbers of bifid-tailed *cercariæ*, which escape into the surrounding water. A lens is required to detect these tiny creatures in natural waters, though experimentally in a test-tube their appearance to the naked eye is that of a swarming mass of minute white hairs. The infected snail goes on discharging them for several weeks, and they constitute the infective agent, as they can and do penetrate the unbroken skin, and pass even more readily through the buccal mucous membrane when taken in drinking water. Once in the blood stream, they reach the liver to develop into adult male and female bilharzia worms, the process occupying about two months for completion, and thereafter the well-known human sexual cycle begins for the females to produce their typically spined eggs, which are passed with their human host's excreta. In the absence of man, however, the *cercariæ* can enjoy a separate existence for only

**Bilharziasis and its History**—(*continued*).

forty-eight hours, and then, unless they gain access to the human system, they die.

### Prophylactic Measures.

Thus it is possible to frame a preventive scheme of considerable practical efficiency. In the first place, any form of personal contact with possibly infected waters must be avoided. Taking the area in question, the Nile, its canals and irrigation channels, the waters of swamps and marshes, and in fact any *permanent* collection of water, natural or artificial, in which the snail hosts may be found, must be treated as dangerous; but in organised situations, obviously from the nature of the infective agent, there are certain easily maintained counter-measures. Boiling of water is a radical method, and can be adopted by the individual, as also the 16-grain water-purifying tablets, two in one quart; but from the point of view of towns and armies the most valuable fact is that, if raw water be stored for more than forty-eight hours before use and great care be taken, by means of gauze or coarse filters, to exclude from the tank all possibly infected molluscs, then the *cercariæ* will have perished meanwhile. Filtration through shallow sand is not reliable, and probably the apparent efficiency of filtration on the large town scale is due, actually, to the length of time intervening between collection and consumption of the water. It was found in Egypt that ordinary routine water chlorination does not necessarily protect, and, owing to rapid deterioration of chlorinated lime in the heat, the difficulty of producing sufficient chlorine concentration and the necessity of rendering a highly chlorinated water potable made other methods preferable. Bathing water gives rise to a simpler problem. Immediate safety is produced by adding undiluted Army cresol in a dilution of one in 10,000, but again we must note that, if the water can be kept even overnight, one in 90,000 is sufficient, so rapidly do the *cercariæ* lose their vitality.

### The Disease among Soldiers.

Dr. Fairley divides the phases observed during sundry outbreaks among troops in Egypt into (*a*) a toxæmic stage occurring four to ten weeks after infection, and (*b*) a much later stage of localised disease, characterised in the case of *B. hæmatobia* by vesical and in *B. mansoni* by intestinal symptoms. Most observers agree with Lawton's description of an outbreak among the Australian troops in 1916. The toxæmic stage, after an incubation period of four to eight weeks, becomes apparent with abdominal pain, hepatic and splenic enlargement, pyrexia lasting ten days to eight weeks, bronchitis urticaria, diarrhœa, and marked eosinophilia. The infection was here with *B. mansoni*, and Fairley describes an exactly similar condition with pure *hæmatobia* infection. He has carried out a number of experimental infections in apes, and finds good evidence that these initial symptoms are definitely toxic in origin. Also an antibody can be demonstrated in the peripheral blood of infected persons, by means of a specific complement-fixation test exactly analogous to the Wassermann reaction, which, together with the eosinophilia and excess of eosinophil myelocytes in the bone marrow, form strong evidence of specific toxic action. In any case, stage (*a*) is transient, marked essentially by pyrexial and urticarial symptoms, and following it, in spite of the fact that ova are being passed into the excreta the whole time, comes a latent period lasting anything between six weeks to two and a-half years before obvious localised symptoms of bilharziasis are noted.

### Later Symptoms and Treatment.

It is impossible in the space at our disposal to describe in any detail the later manifestations of this disease, but one may point out that, except in such secondary states as sepsis and malignancy, which frequently arise in old-standing cases, the symptoms are all attributable to the mechanical effect of the *spined ova*. They are deposited according to species, chiefly in fine terminal vessels of the bladder and rectum, and, working their way to the surface in order to escape, act simply as so many minute foreign bodies. In making these journeys the ova may take many months, and apparently the parent worm lives for long periods, instances being known where typical bilharziasis, with ova in the excrement, has persisted for ten years without known opportunity of re-infection. Secondary states—abscesses, fistulæ, pyelitis, etc.—must be dealt with by customary methods, but, owing to the mode of irritation, it is suggested that if one is able to kill the worm, and at least sterilise the ova deposited in the tissues, it might fairly be said that the patient is cured. Such is the suggestion made by Dr. Christopherson, director of the civil hospitals at Khartoum and Omdurman, and this authority, on the strength of experience gained in treating the disease by intravenous antimony tartrate as a routine method since May 1917, states:

> I think we are justified in saying that it not only kills first the parasite *in situ*, but that later it kills the embryo in the ova deposited in the terminal vessels, and sterilises the ova so deposited, and that the patient is cured not only of his bilharzia parasites but that he ceases to be a bilharzia carrier, and cannot propagate the disease.

In outline, this treatment requires use of a solution of ½ gr. antimony tartrate in 20♏ distilled water, which before injection is diluted with once or twice its volume of saline, the method being applied at first daily, then on alternate days, etc., microscopic observation of the urine or fæces being kept throughout. For greater detail one must refer the reader to Dr. Christopherson's own excellent descriptions, and, if similar success in treatment of bilharziasis is met with by the large number of men whom we know to be now employing his methods, there is no doubt that, apart from the peculiar interest of its causal parasite, this disease will demand attention as one which is now complete—at least, complete according to our present standards—in ætiology, symptomatology, prophylaxis, and cure.

